# Antiretroviral treatment in adult ethnobotanical intravenous drug users recently infected with HIV

**DOI:** 10.1186/1471-2334-14-S4-O10

**Published:** 2014-05-29

**Authors:** Ionuț Cristian Popa, Simona Erscoiu, Cristina Pătru, Olivia Burcoş, Tatiana Stoicev, Emanoil Ceauşu, Nicoleta Pîrvu, Denis Oncel, Raia Loghin, Ana-Maria Mehedinți

**Affiliations:** 1 Clinical Hospital of Infectious and Tropical Diseases “Dr. Victor Babeş”, Bucharest, Romania

## 

During the last few years in Romania, increased numbers of new HIV infections among ethnobotanicals intravenous drug users (eIVDU) were observed. Although most cases have relatively good immunological status, some patients reclaim antiretroviral (ARV) treatment because of very low CD4 count, severe comorbidities and/or pregnancies despite difficulties related to their adherence.

Retrospective analysis of 32 patients, eIVDU, treated with ARV in our hospital between 1^st^ January 2010 and 31^st^ January 2014.

From all 32 patients, 27 are males (84.4%). The main reason for starting ARV treatment was represented by severe AIDS-defining illness (71.4%) related to immunodepression with CD4 count below 100 cells/cmm 11 cases (34.3%) and one pregnancy. In our study group the mean CD4 count at baseline was of 110.1 cells/cmm (median CD4 102.5 cells/cmm). 24 patients (75%) received at first triple therapy including NNRTI (10 cases with nevirapine, 14 cases with efavirenz), the other 8 patients (25%) received from the beginning regimens with protease inhibitors. 7 patients from the first group were then switched to protease inhibitors, mostly after completing tuberculostatic treatment, while 2 patients from the second group were switched to integrase inhibitors because of potential drug-drug interactions involving rifampin. All patients in our study were coinfected with HCV, 9 patients (28.9%) had also HBV, and 15 patients (46.8%) had active tuberculosis. Only 15 patients were truly adherent to ARV (43.7%); we registered 5 cases of abandon and one death. From all 5 patients who discontinued treatment 3 were social cases; from all 28 patients who are currently treated 20 have good family support (71.4%) and 6 (21.4%) receive psychological counseling and methadone substitution therapy. Mean value of CD4 count at 1 month of HAART therapy was of 225.8 cells/cmm, probably related to ARV treatment received correctly during admission in our clinic, but this value decreased to 213.3 cells/cmm at 3 months, probably related to poor compliance at home.

Despite the fact that they are recently infected with HIV, increasingly more eIVDU patients are in need of ARV treatment, because mainly of rapid immunodepression and comorbidities associated with AIDS. Family support as well as psychological counseling and substitution therapy with methadone are mandatory for achieving clinical success with HAART, while lack of adherence is the principal cause for immunological and virological failure. Tuberculosis with both pulmonary and extrapulmonary involvement is the principal complication associated with AIDS, involving almost half of the eIVDU in our country.

